# High-Yield Production of Chimeric Hepatitis E Virus-Like Particles Bearing the M2e Influenza Epitope and Receptor Binding Domain of SARS-CoV-2 in Plants Using Viral Vectors

**DOI:** 10.3390/ijms232415684

**Published:** 2022-12-10

**Authors:** Eugenia S. Mardanova, Roman Y. Kotlyarov, Maya D. Stuchinskaya, Lyudmila I. Nikolaeva, Gergana Zahmanova, Nikolai V. Ravin

**Affiliations:** 1 Institute of Bioengineering, Research Center of Biotechnology of the Russian Academy of Sciences, 119071 Moscow, Russia; 2 N.F. Gamaleya National Research Centre of Epidemiology and Microbiology, Ministry of Health, 123098 Moscow, Russia; 3 Department of Plant Physiology and Molecular Biology, University of Plovdiv, 4000 Plovdiv, Bulgaria; 4 Center of Plant Systems Biology and Biotechnology, 4000 Plovdiv, Bulgaria

**Keywords:** hepatitis E virus, RBD of SARS-CoV-2, M2e peptide, influenza A virus, plant-produced vaccine, virus-like particle

## Abstract

Capsid protein of Hepatitis E virus (HEV) is capable of self-assembly into virus-like particles (VLPs) when expressed in *Nicotiana benthamiana* plants. Such VLPs could be used as carriers of antigens for vaccine development. In this study, we obtained VLPs based on truncated coat protein of HEV bearing the M2e peptide of Influenza A virus or receptor-binding domain of SARS-CoV-2 spike glycoprotein (RBD). We optimized the immunogenic epitopes’ presentation by inserting them into the protruding domain of HEV ORF2 at position Tyr485. The fusion proteins were expressed in *Nicotiana benthamiana* plants using self-replicating potato virus X (PVX)-based vector. The fusion protein HEV/M2, targeted to the cytosol, was expressed at the level of about 300–400 μg per gram of fresh leaf tissue and appeared to be soluble. The fusion protein was purified using metal affinity chromatography under native conditions with the final yield about 200 μg per gram of fresh leaf tissue. The fusion protein HEV/RBD, targeted to the endoplasmic reticulum, was expressed at about 80–100 μg per gram of fresh leaf tissue; the yield after purification was up to 20 μg per gram of fresh leaf tissue. The recombinant proteins HEV/M2 and HEV/RBD formed nanosized virus-like particles that could be recognized by antibodies against inserted epitopes. The ELISA assay showed that antibodies of COVID-19 patients can bind plant-produced HEV/RBD virus-like particles. This study shows that HEV capsid protein is a promising carrier for presentation of foreign antigen.

## 1. Introduction

Various expression systems, such as bacteria, yeast, plants, insect or animal cells, can be used for the production of recombinant proteins for medical and veterinary purposes. Plants can become a competitive “biofactory” for such purposes [1,2,3]. The advantages of plants include the low cost, ease of scaling, the presence of eukaryotic post-translational modifications, as well as the absence of common pathogens in animals and plants, which makes preparations obtained in plants safe. A high level of production of recombinant proteins in plants can be achieved through the use of agroinfiltration-based transient expression systems employing recombinant plant viral vectors [4,5,6]. Such expression platforms can be easily and quickly developed for a new target; they are easily scalable, cost-effective and enable high levels of protein expression in several days. A number of studies have demonstrated the feasibility of plant-based systems for the production of vaccines and other biopharmaceuticals [3,7,8,9,10,11].

Medicago (Quebec, QC, Canada) has submitted a plant-produced vaccine against seasonal influenza, which has passed three stages of clinical trials, for consideration by regulatory authorities [12]. Medicago successfully produced virus-like particles (VLPs) of the 2019 novel coronavirus just 20 days after obtaining the SARS-CoV-2 gene [13]. This company registered the first plant-based vaccine COVIFENZ^®^ COVID-19 Vaccine (plant-based virus-like particles, recombinant, adjuvanted) in 2022 [14].

Plant biofactories may become an instrument for a rapid response to pandemic threats. Therefore, plant platforms can be used to create vaccines against different infections. Influenza A is a widespread viral infection in humans and animals. New strains of the virus appear every one to two years due to the high variability of viral surface proteins, such as hemagglutinin and neuraminidase [15]. The use of conserved virus proteins would allow the development of “universal” vaccines that could be efficient against a wide range of influenza strains. The extracellular domain of the transmembrane protein M2 (M2e), 23 a.a. in size, is a promising conserved antigen of the influenza A virus [16]. Its sequence is highly conserved among human strains and has only a few amino-acid replacements in influenza A strains of animal origin [17,18,19]. The disadvantage of this peptide is its low immunogenicity [20]; however, it becomes highly immunogenic and provides protection against infection when fused to an adjuvant protein or carrier VLPs [21]. Several candidate vaccines based on the M2e peptide fused to various carriers have been developed for the expression in plants [22]. Proteins such as flagellin [23,24], Cucumber mosaic virus coat protein [25], Cowpea mosaic virus (CPMV) particles [26], Alternanthera mosaic virus capsid protein [27], Tobacco mosaic virus [28,29], Human papillomavirus L1 protein [30], Hepatitis B virus core protein [31,32,33], Zera@ Tag [34], β-glucuronidase [35], and Ricin Toxin B Chain [36] were used as carriers of M2e.

Since the end of 2019, SARS-CoV-2 has been spreading from the city of Wuhan, Hubei province of China, around the world. At present, the virus has tended to become a seasonal infection, thus vaccine development is an actual task. Spike glycoprotein (S) of SARS-CoV-2 is the most commonly used vaccine candidate. Cryo-electron microscopy made it possible to recognize the receptor-binding domain (RBD) in the structure of the S-protein, which directly binds to the angiotensin-converting enzyme 2 (ACE2) receptor [37,38,39]. Moreover, most of the potent monoclonal antibodies to SARS-CoV-2 target the RBD [40].

RBD was expressed in plants alone [41,42,43] with a maximal purification yield of about 20 μg per gram of fresh leaf tissue or as a fusion protein [44,45,46]. Plant-produced RBD antigens elicited high titers of antibodies with a potent virus-neutralizing activity [43]. RBD fused to flagellin of *Salmonella typhimurium* was transiently expressed in plants at the purification level of about 100 μg/g fresh weight and such fusion could be used for the development of intranasal vaccine [45]. Plant-produced RBD fused with the Fc fragment of human IgG1 was immunogenic in mice and non-human primates [44]. The expression level of plant-produced SARS-CoV-2 RBD-Fc was 25 μg/g fresh weight. The vaccine candidate consisted of the RBD fused to a human IgG1 Fc domain and conjugated to a modified tobacco mosaic virus (TMV) nanoparticles induced strong antibody responses in mice [46]. Kentucky BioProcessing is developing a recombinant subunit RBD-based vaccine in plants and has already started phase 1–2 clinical trials (NCT04473690). In other studies, it was shown that simultaneous transient expression of envelop (E), membrane (M), and S proteins in *Nicotiana benthamiana* resulted in VLP formation [47,48]; as well as the co-expression of M, E and nucleocapsid (P) [49].

VLPs are highly immunogenic structures and can induce protective immunity [50]. The use of VLPs as carriers provides the possibility to increase the immunogenicity of the foreign antigens linked to them. As a carrier protein for VLP production, we used truncated coat protein of Hepatitis E virus (HEV) (genotype 3). HEV virions are non-enveloped icosahedral particles of 27–32 nm in size, resulting from the self-assembly of the capsid protein [51]. The capsid protein, encoded by the second open reading frame (ORF2) located at the 3́ terminus of the genome, comprises 660 amino acids. It is a major antigenic and immunogenic protein, containing epitopes responsible for the induction of virus-neutralizing antibodies [52,53,54,55]. The shorter version of the capsid protein consisting of a.a. residues 110 to 610 was able to form virus-like particles when expressed in *N. benthamiana* plants [56,57].

The reconstructed VLPs displayed a T1 icosahedral particle composed of 60 copies of truncated pORF2 with a diameter of 27 nm [58]. The crystal structures were reported for genotype 1 T1 VLPs [59], genotype 3 T1 VLPs [60], and genotype 4 T1 VLPs [61], revealing that pORF2 is composed of three domains, the S domain, M domain, and P domain comprising the amino acid residues 129–319, 320–455, and 456–606, respectively, for genotype 3 [60]. The reconstructed structures for these genotypes are in good agreement with each other [62].

The S-domain assembles into a stable icosahedral shell, while the P-domain protrudes as a surface spike, and is involved in virus–host interactions and contains neutralization epitopes [61]. On top of the surface spike, there are three highly exposed large loop insertions (a.a. 482–490, 550–566, and 583–593) that may play an important role in antigenicity determination (genotype 4) [61]. It was shown that anti-HEV monoclonal antibodies (Fab224) bound to the protruding domain of the capsid protein at the lateral side of the spikes [62] (genotype 1). The antibodies recognize a conformational epitope, and its binding site covers a surface composed of three loops, including a.a. 470 to 493, 539 to 569, and 581 to 595 [62]. Residues Glu479, Asp481, Thr484, Tyr485, Ser487, Tyr532, Ser533, and Lys534 were in close contact with the Fab molecule [62]. It was found that double mutations that changed residues Glu479 and Lys534 or Tyr485 and Ile529 to alanine selectively abrogated pORF2′s reactivity with neutralizing antibodies [63]. If an insertion can be placed at the antibody-binding site at the P-domain, the chimeric VLP will not be a target for acquired immunity after infection with HEV.

Previously, six insertion sites for 11 a.a. long B cell epitope tag were selected according to restriction enzyme sites located either internally (four sites) or at the N or C terminus of pORF2. The internal sites are located after residues Ala179, Arg366, Ala507, and Arg542. Fusion proteins carrying insertions at sites Ala179 and Arg336 completely failed to produce VLPs, and insertions at Ala507 and Arg542 greatly reduced VLP production [64]. Crystal structure data revealed that the spatial position of these sites is disadvantageous since the C terminus is exposed on the surface of VLPs, while the N terminus points toward the VLP center. Therefore, insertions at the termini do not inhibit VLP assembly; however, the C terminus is more suitable for tethering bulky foreign antigenic sequences [64]. The binding site of Fab224 appeared to be distinct from the location of the inserted B-cell tag, suggesting that the chimeric VLP could elicit immunity against both HEV and an inserted foreign epitope [62].

The computational modeling of the full-length HEV coat protein (genotype 3) revealed three regions which are most suitable for peptide insertion (a.a. 480–490, 550–565, and 580–590) and correspond to three loops on the surface spike [65]. The 15 a.a. long antigenic p18 peptide from the V3 loop of HIV-1 gp120 (RIQRGPGRAFVTIGK) was inserted after residue Tyr485 on the P-domain of HEV CP [66]. The fusion protein was able to form VLPs which did not react with anti-HEV antibodies. Thus, the residue Tyr485 is a promising candidate for insertion of a short peptide without interfering with either tertiary structure folding or capsid assembly [66].

Previously, we reported the creation of a hybrid HEV capsid protein consisting of 110–610 a.a. with influenza M2e peptide inserted at position Gly556 [56]. Such fusion protein was successfully expressed in plants and formed VLPs, but they appeared to be heterogeneous in size and shape [56]. The HEV capsid has not previously been used as a carrier of the RBD peptide.

In this study, we constructed hybrid proteins based on the truncated HEV capsid protein (110–610 a.a.) with M2e and RBD peptides inserted at the Tyr485 position. Both proteins were transiently expressed in plants using a viral-based expression system and were found to form nanosized VLPs.

## 2. Results

### 2.1. Viral Vectors for the Expression of HEV Capsid Proteins Comprising M2e and RBD Peptides at the Tyr485 Position

**Figure 1:**
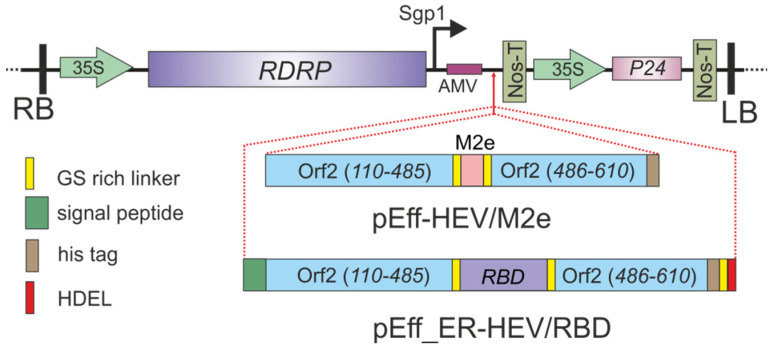
Scheme of the expression vectors pEff-HEV/M2 and pEff_ER-HEV/RBD. RDRP, RNA-dependent RNA polymerase of PVX; Sgp1, the first promoter of the subgenomic RNA of PXV; AMV, the leader sequence of RNA 4 of alfalfa mosaic virus; 35S, promoter of the cauliflower mosaic virus RNA; NosT, terminator of the nopaline synthase gene from *A. tumefaciens*; P24, gene of silencing suppressor from grapevine leafroll-associated virus-2; LB and RB are the left and right borders of T-DNA. Schematic diagrams of the chimeric HEV/M2 and HEV/RBD proteins are shown at the bottom of the figure.

The pEff viral vector [33] was used to express the target proteins HEV/M2e and HEV/RBD in *N. benthamiana* plants. To create HEV-M2e fusion, the M2e peptide was genetically inserted at the Tyr485 position of HEV capsid protein. Flexible glycine-rich linkers GGGSG were inserted to flank M2e and facilitated folding of the hybrid proteins. A 6-histidine tag was added at the C-terminus of HEV/M2 protein to enable its purification by metal affinity chromatography (Figure 1). No sequences targeting recombinant proteins to a specific cellular compartment were used.

For the expression of HEV/RBD fusion, the pEff expression vector was first modified to encode the signal peptide (MIMASSKLLSLALFLALLSHANS) from *Phaseolus vulgaris* at the N-terminus and the endoplasmic reticulum retention signal sequence HDEL at the C-terminus of the target protein. These signal sequences were included for targeting the recombinant protein to the endoplasmic reticulum (ER) and its retention in this compartment. Additionally, eight histidine tag and GGS linkers were inserted between the target protein and the HDEL sequence (Figure 1). The RBD region (spanning from amino acid 319 to 524) flanked by GGGSG linkers was inserted at the Tyr485 position of HEV capsid protein (Figure 1), The strategy of the ER targeting was previously applied for the expression of RBD in plants [42,43]. Such a method can influence the expression efficiency of the recombinant proteins, its stability, and solubility.

To obtain recombinant viral vectors pEff-HEV/M2e and pEff_ER-HEV/RBD allowing expression of HEV/M2e and HEV/RBD fusions, the corresponding genes were cloned in the pEff vector (Figure 1). Recombinant viral vector pEff_HEV 110-610_his was used for expression of the control HEV capsid [57].

### 2.2. Expression and Purification of HEV/M2e and HEV/RBD Proteins

Recombinant binary vectors were introduced into *A. tumefaciens* strain GV3101 by electroporation. The obtained strains were used for the infiltration of the leaves of *N. benthamiana* plants. Leaf tissues for isolation of protein samples were harvested four days after agroinfiltration.

**Figure 2:**
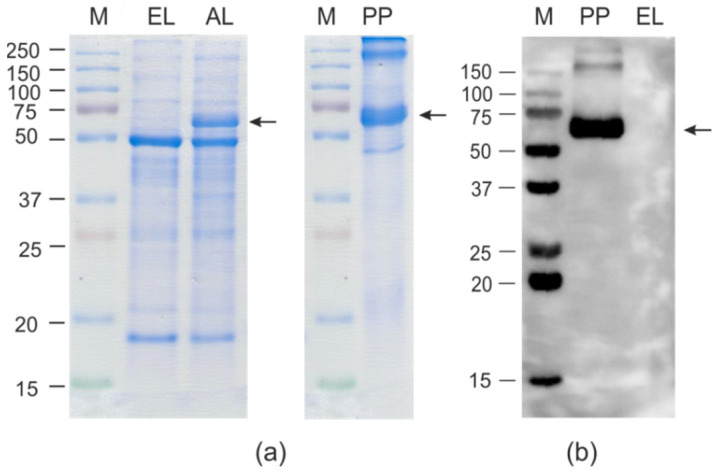
Expression of HEV/M2e protein in *Nicotiana benthamiana* plants. Coomassie brilliant blue-stained gel (**a**) and Western blot (**b**) of proteins isolated from *N. benthamiana* plants. M, molecular weight marker (sizes are shown in kD); EL, total proteins isolated from the non-infiltrated leaf; AL, total proteins isolated from leaf infiltrated with pEff-HEV/M2e; PP, purified HEV/M2e protein. Western blotting was performed using anti-M2e antibodies. The position of HEV/M2e protein (calculated molecular weight 56 kD) is shown by an arrow. The larger bands in lane PP are likely the result of disulfide-bond-mediated aggregation of the M2e peptides.

**Figure 3:**
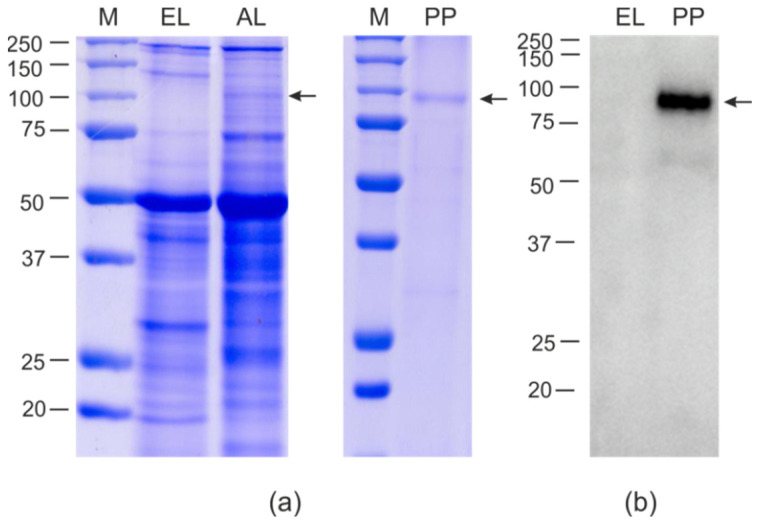
Expression of HEV/RBD protein in *Nicotiana benthamiana* plants. Coomassie brilliant blue-stained gel (**a**) and Western blot (**b**) of proteins isolated from *N. benthamiana* plants. M, molecular weight marker (sizes are shown in kD); EL, total proteins isolated from the non-infiltrated leaf; AL, total proteins isolated from leaf infiltrated with pEff_ER-HEV/RBD; PP, purified HEV/RBD protein. Western blotting was performed using anti-RBD antibodies. The position of HEV/RBD protein (calculated molecular weight 81 kD) is shown by an arrow.

An SDS-PAGE analysis showed that HEV/M2e was efficiently synthesized in plants accounting for about 10% of the total protein, corresponding to 300–400 μg/g of fresh leaf tissue. Nearly the same level of expression was previously reported for HEV without foreign peptides [56,57]. Therefore, the M2e insertion did not decrease the efficiency of expression of HEV capsid (Figure 2). The hybrid protein HEV/RBD was expressed in *N. benthamiana* less efficiently, at the level of about 1–2% of the total protein (80–100 μg/g), according to SDS-PAGE estimates (Figure 3).

To purify the recombinant proteins, the biomass of agroinfiltrated leaves was collected on the 4th day after infiltration. Protein purification was performed using metal-affinity chromatography under native conditions. After purification, the protein samples were dialyzed against phosphate-buffered saline (PBS).

Samples of purified HEV/M2e and HEV/RBD proteins were characterized using SDS-PAGE and Western blotting (Figure 2 and Figure 3). The final yields of HEV/M2e and HEV/RBD after purification were up to 200 μg and 20 μg per 1 g of green leaf biomass, respectively. Both HEV/M2e and HEV/RBD proteins were specifically revealed in Western blot analysis with the antibodies to M2e and RBD, respectively.

The calculated molecular weight of the HEV/RBD protein without N-terminal signal peptide is 81 kD, however, it migrated slowly in SDS-PAGE, although this protein is not highly acidic or hydrophobic. A similar phenomenon was observed for RBD fusion with flagellin [45], and for the HEV capsid itself [56]. Being targeted to the endoplasmic reticulum in plant cells, the HEV/RBD protein is probably glycosylated and thus is expected to move slowly, as reported previously for another variant of the RBD peptide [43].

### 2.3. Recombinant Proteins HEV/M2e and HEV/RBD form Virus-Like Particles

**Figure 4:**
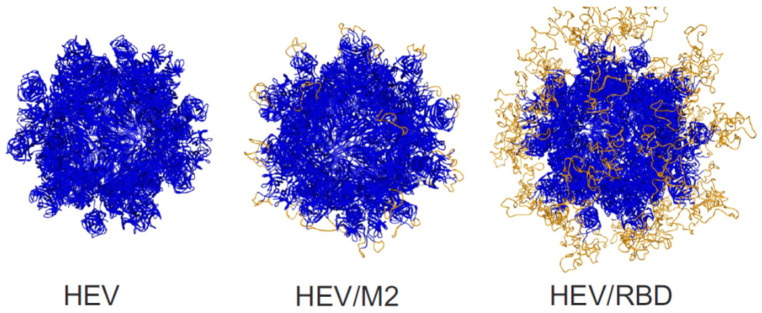
The 3D modeling of virus-like particles formed by HEV ORF2 capsid, HEV/M2e, and HEV/RBD proteins by SWISS MODEL.

The computational modelling was used to predict the spatial structures of HEV, HEV/M2e and HEV/RBD molecules. The model was built using the homology modelling web-based integrated service, SWISS-MODEL [67,68,69,70]. This software is based on predicting the structure of the target by utilizing the information from homologous proteins which structure is already experimentally determined. The 3D structures of HEV, HEV/M2e, and HEV/RBD were successfully predicted. Each molecule was predicted to assemble into VLPs of 60 monomers. RBD and M2e peptides were predicted to be exposed on the surface of VLPs (Figure 4).

**Figure 5:**
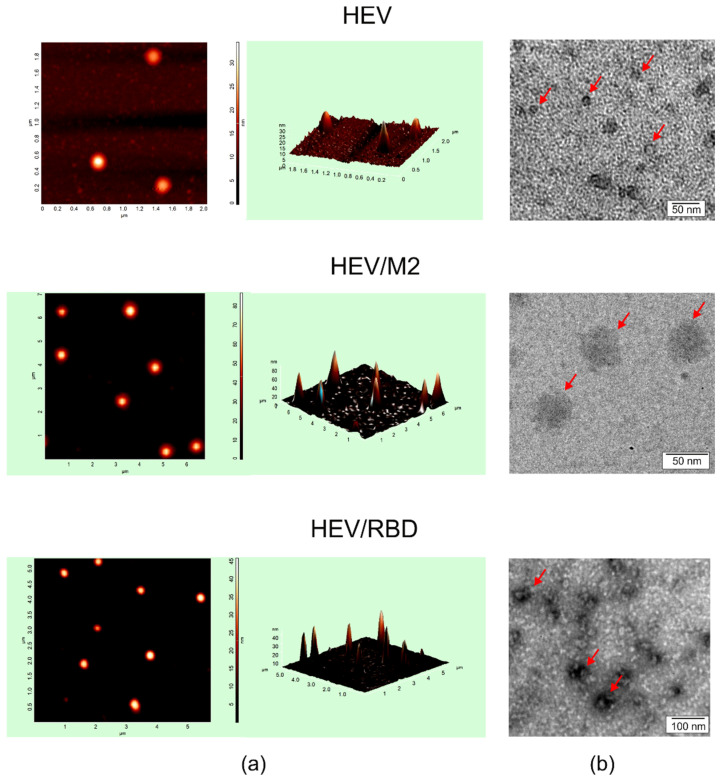
Analysis of virus-like particles formed by HEV ORF2 capsid, HEV/M2e, and HEV/RBD proteins by atomic force microscopy (**a**) and transmission electron microscopy (**b**). Arrows indicate virus-like particles.

The assembly of HEV/M2e and HEV/RBD proteins into nanosized structures was experimentally analyzed by dynamic light scattering, electron, and atomic force microscopy. Particulate structures were observed by all three methods (Figure 5).

The size of HEV/M2e particles estimated by electron microscopy was about 40 nm, atomic force microscopy revealed about 60 nm structures. According to dynamic light scattering measurements, the samples contained particulate structures about 50 nm in size and 200–300 nm aggregates. Examination of the HEV/RBD samples by electron and atomic force microscopy revealed VLPs with size of 30–40 nm, while dynamic light scattering detected ~40 nm particles and aggregates 200–300 nm in size (Table 1). In comparison, the HEV 110-610 capsid protein without any fusion partners formed smaller particles with an estimated size of 18–26 nm (Table 1 and [71]).

### 2.4. VLPs Formed by Recombinant Protein HEV/RBD Specifically Interacts with Sera of COVID-19 Patients

**Figure 6:**
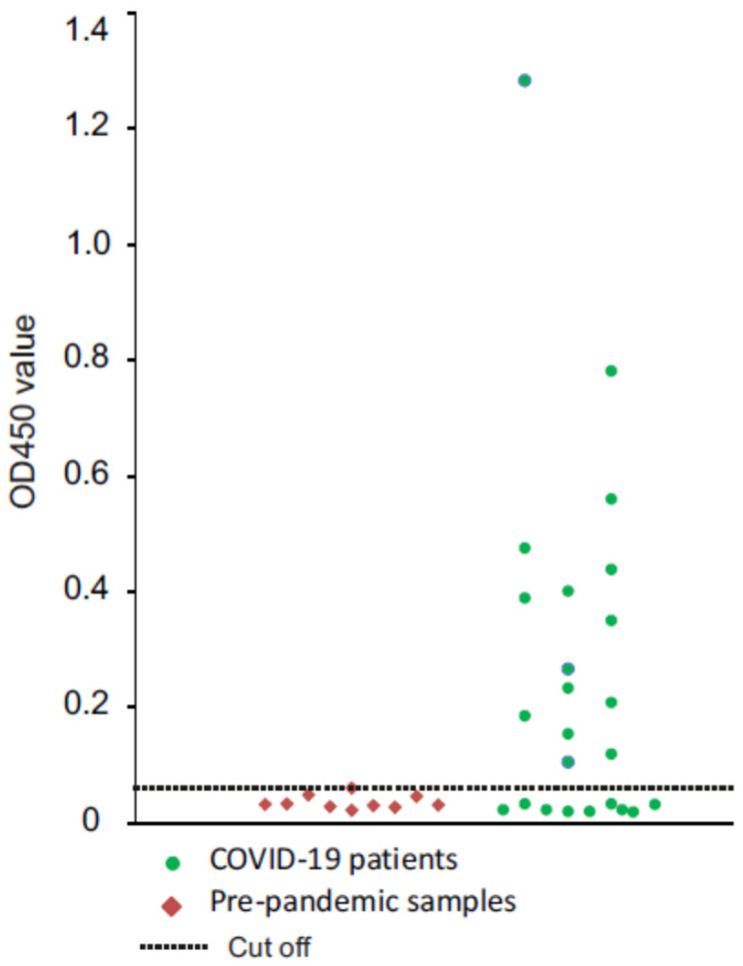
Plant-derive HEV/RBD detects IgG in serum samples from COVID-19-recovered patients (*n* = 24). Results are presented as optical density value (OD) of analyzed sera. The serum dilution was 1:80. A cut-off for positivity (0.059, indicated by a dotted line) was determined as two standard deviations above the mean optical density of pre-pandemic sera (*n* = 10), the serum dilution was 1:80.

To examine whether the HEV/RBD could be recognized by human antibodies, we tested human sera from 24 recovered PCR-positive COVID-19 patients by ELISA. As a negative control, 10 serum samples from individuals collected prior to the pandemic (2016–2018) were analyzed in parallel. All serum samples were negative in ELISA with HEV as an antigen. The ELISA assay showed that 15 of 24 serum samples of COVID-19 patients were positive in ELISA with HEV/RBD as antigen, while all control samples appeared to be negative (Figure 6). The assay showed that the antibodies produced in COVID-19 patients can bind plant-derived HEV/RBD VLPs, suggesting that the RBD are accessible for the antibodies and possibly are exposed on the surface of the HEV particle.

## 3. Discussion

The use of virus-like particles is a convenient and effective tool for the positioning of the foreign epitopes in the development of vaccines against various diseases. Hepatitis E virus can be a basis for such VLPs. HEV virions are non-enveloped icosahedral particles of 27–32 nm in size, resulting from the self-assembly of ORF2 capsid protein. 

The insertion of peptides at the surface antigenic site could allow VLPs to escape pre-existing anti-HEV humoral immunity in people previously infected with HEV. The binding site of anti-HEV monoclonal antibody HEP224 is composed of three surface loops around residue Tyr485 of HEV ORF2 [62]. In the study of Pitchanee Jariyapong [66] it was shown that insertion of p18 peptide of HIV-1 (RIQRGPGRAFVTIGK) at Tyr485 disrupted the interaction of these loops leading to conformational rearrangement. The insertion of p18 inhibited the binding of antibodies to the carrier, but did not completely shield the antigenic domain [66]. We have inserted the M2e peptide, the extracellular domain of transmembrane protein M2 of influenza A virus, and RBD, receptor-binding domain of SARS-CoV-2 spike glycoprotein, at the Tyr485 position of HEV ORF2, the most promising epitope insertion site.

An important advantage of HEV 110–610 capsid as a carrier is the possibility of its production in plants at a high level. Application of self-replicating viral vector pEff allowed the expression of HEV/M2 in plants at the level of 300–400 μg/g of fresh leaf biomass, and the purification yield reached 200 μg/g. These values are approximately tenfold higher than those achieved using a similar expression system for the production in plants of M2e-bearing VLPs based on the core antigen of the hepatitis B virus [32]. Notably, insertion of M2e did not reduce the expression level relative to unmodified HEV 110–610 capsid and did not affect the solubility of the hybrid protein. Moreover, the insertion of M2e epitope did not interfere with VLP formation. The antigenicity of the M2e peptide was maintained and Western blot with anti-M2e antibodies showed a strong recognition of the HEV/M2 chimeric protein.

Since the HEV capsid can be expressed in plants at a high level, we used it as a carrier for the RBD. In order to facilitate expression in plant cells, we targeted the HEV/RBD protein to the endoplasmic reticulum by adding ER-targeting signal peptide at its N-terminus and ER retention signal HDEL at the C-terminus. Such targeting strategy was previously used for the RBD expression in plants [42,43]. Due to such modification, about 20% of HEV/RBD remained in the soluble fraction, while the same protein without targeting signals was completely insoluble. The protein was expressed up to 100 μg per gram of green leaf biomass, and after purification under native conditions the yield was about 15–20 μg per 1 g, a value sufficient for commercialization of about plant-produced COVID-19 vaccine candidates [43]. 

Nevertheless, the introduction of RBD affects both the solubility of the hybrid protein and the level of its expression. The insertion of additional sequences can modify the properties of the fusion protein, as well as decrease the efficiency of its expression and solubility, affect the ability to form nanoparticles, etc. (for example, [24,72]). This problem is particularly evident in the case of long and/or hydrophobic insertions such as the RBD peptide comprising 80 hydrophobic of a total of 205 a.a. The RBD peptide also contains seven cysteine residues, which can cause the formation of disulfide bonds and protein aggregation. For example, plant-expressed hybrid protein consisting of RBD fused to flagellin of *Salmonella typhimurium* appeared to be fully insoluble and was purified at the level of about 100 μg per 1 g of green leaf biomass, whereas empty flagellin was soluble and its purification yield was three times higher [23,45].

The efficiency of HEV/RBD expression could be enhanced by the codon optimization for *N. benthamiana*. Insertion of longer flexible glycine-rich linkers at the junction points between RBD and HEV sequences may facilitate folding of the fusion protein and enable its production in a soluble form. Finally, co-expression of HEV/RBD and a capsid without an insert may allow the production of chimeric VLPs in which only a fraction of the monomers will contain an RBD insert. Although the density of presentation of the RBD on the particle will decrease, it can be expected that the better spatial separation of the RBD peptides will reduce aggregation and increase the stability of VLPs. 

One of the actively developing areas in immunology is the design of vaccines based on proteins that can self-assemble into nanosized particles [73]. The use of such artificial virus-like particles significantly increases the immunogenicity of peptides exposed on their surface [74,75]. Both HEV/M2 and HEV/RBD proteins formed virus-like nanoparticles of spherical shape in vivo when expressed in plant cells. Moreover, such recombinant VLPs will probably induce an immune response to the carrier and therefore can be potentially used to develop bivalent vaccines additionally against HEV. This is an important additional benefit since HEV infection is a worldwide disease and the primary cause of acute viral hepatitis in the world, with an estimated 20 million cases every year [76].

## 4. Materials and Methods

### 4.1. Expression Vectors

Potato virus X (PVX)-based vector pEff was used for the expression of the recombinant proteins [33]. This vector contains the 5′-untranslated region of the PVX genome, the gene for RNA-dependent RNA polymerase enabling the replication of the vector in a plant cell, promoter of the first viral subgenomic RNA, the AMV translation enhancer (5′-nontranslated region of RNA 4 of the alfalfa mosaic virus), the *gfp* gene flanked by unique *Asc*I and *Sma*I sites that can be replaced by the gene of the interest, and the 3′-untranslated fragment of the PVX genome. pEff also contains expression cassette for P24 suppressor of silencing from grapevine leafroll-associated virus-2. Both expression cassettes are located between the 35S promoter of the cauliflower mosaic virus and the Nos-T terminator of the nopaline synthase gene from *A. tumefaciens*. All these genetic elements are located in the T-DNA region of a binary vector that could be maintained in both *Escherichia coli* and *Agrobacterium tumefaciens*. This pEff vector was previously used for the fast high-level expression of recombinant proteins in plant cells [5,24,45,56,77].

### 4.2. Gene Cloning and Construction of Plasmids for Expression of HEV/M2e

As a basis for plasmid constructions, we used HEV 110-610 gene (amino acids from 110 to 610 of ORF2), described in [57]. The HEV 110-610 gene was amplified using HEV110_Asc-F (TAG GCG CGC CAT GGG TAT GGC TAC TTC TCC TG)/HEV_Sma-R (ATC CCG GGC TAA GCA AGA GCA GAG TGA GGA G) primers and recombinant pEAQ vector containing HEV 110-610 sequence [56] as a template and cloned into pGEM vector (Promega, Madison, WI, USA). To introduce the restriction sites for *Xho*I and *Sna*I into the HEV 110-610 gene, the obtained plasmid was used as a template for PCR amplification with primers F_HEV_485_Sna/Xho (ATC TCG AGT ACG TAG GTT CTA GCA CCA ACC CTA T) and R_HEV_485_Xho (ATC TCG AGG TAG GTG GTC TGA TCG TAC T). The obtained DNA fragment was digested with *Xho*I and circularized by self-ligation; the resulting plasmid was named pGEM-HEV 110-610_ *Xho*I/*Sna*I.

The DNA sequence encoding M2e flanked by GGGSG linkers was amplified by PCR using primers M2eh_GS_Xho_F (ATC TCG AGG GAG GTG GAT CTG GAT CTC TTC TGA CCG AGG TGG A) and M2eh_GS_Sna_R (TAT ACG TAT CCA GAA CCA CCT CCA TCG GAG CTA TCG TTG CAT C) from pEff-M2 HEV 110–610 template [56]. The PCR fragment was cloned into pGEM-HEV 110-610_ *Xho*I/*Sna*I at *Xho*I and *Sna*I sites. 

A 6-histidine tag was added at the C-terminus of the hybrid proteins to facilitate its purification by metal affinity chromatography. The his-tagged HEV/M2e gene was amplified by PCR using primers HEV110_Asc-F (TAG GCG CGC CAT GGG TAT GGC TAC TTC TCC TG)/HEV-his_R (TAC CCG GGC TAA TGA TGG TGA TGG TGA TGA GCA AGA GCA GAG TGA GGA GCA AG) and cloned into pEff vector at *Asc*I and *Sma*I restriction sites resulting in recombinant pEff_HEV/M2e vector.

PCR amplifications were carried out using Mastercycler^®^ nexus thermocycler (Eppendorf, Hamburg, Germany) and KAPA2G Fast HotStart PCR Kit (KAPA Biosystems, Wilmington, MA, USA). The following PCR conditions were used: 94 °C for 3 min, followed by 40 cycles of denaturation at 94 °C for 1 min, annealing at 55 °C for 40 s, and extension at 72 °C for 2 min; the final extension was at 72 °C for 5 min with subsequent cooling at 10 °C. 

### 4.3. Gene Cloning and Construction of Plasmids for Expression of HEV/RBD

The nucleotide sequence encoding RBD fragment of the S protein (from V319 to V524) of SARS-CoV-2 strain Wuhan-Hu-1 (GenBank QJE37812.1) flanked by GGGSG linkers was obtained by PCR using primers Xho_gs_RBD_F (ATA CTC GAG GGT GGT GGT GGC TCT GTG CAG CCC ACC GAA TCC ATC GT) and Snab_gs_RBD_R (TAT TAC GTA AGA GCC ACC ACC ACC CAC TGT GGC AGG GGC ATG CA), and plasmid pEff-Flg-RBD as a template [45]. The obtained PCR fragment was cloned into the pGEM-HEV 110-610_ XhoI/SnaI vector at XhoI and SnaI restriction sites. 

To accommodate the RBD-coding sequence, we first modified the pEff vector. Synthetic sequence containing *Asc*I restriction site, ER-targeting signal peptide (MIMASSKLLSLALFLALLSHANS), *Cla*I, *Nru*I, *Sac*I and *Sma*I sites, 8 histidine tag, GGS linker, HDEL plant-specific ER retention signal, TAA stop codon and *Stu*I site was cloned into pEff vector at *Asc*I and *Sma*I restriction sites resulting in pEff_ER expression vector. Then, the RBD sequence was amplified by PCR with primers HEV_Sac_F (ATG AGC TCG GTA TGG CTA CTT CTC CTG C), HEV_Sma_R(ΔSTOP) (ATC CCG GGA GCA AGA GCA GAG TGA GGA G) and cloned into pEff_ER at *Sac*I and *Sma*I sites. The resulting expression vector was designated pEff_ER-HEV/RBD. PCR amplifications were performed as described above (Section 4.2).

The constructed vectors pEff-HEV/M2 and pEff_ER-HEV/RBD were isolated from *E. coli* and then electroporated into *A. tumefaciens* strain GV3101. Agrobacteria were grown in LB at 28 °C with kanamycin (50 μg/mL), rifampicin (50 μg/mL), and gentamycin (25 μg/mL).

Expression and purification of the control HEV 110-610 capsid protein was described previously [57].

### 4.4. Agroinfiltration of Nicotiana benthamiana Plants

Plants were grown in a greenhouse under a 16 h daylight regime with additional illumination with full spectrum phyto-lamps until 5–6 true leaves appeared. Recombinant *A. tumefaciens* GV3101 strain with pEff-HEV/M2e or pEff_ER-HEV/RBD vector was grown overnight in a shaking incubator at a temperature of 28 °C. The agrobacterial cells were pelleted by centrifugation for 5 min at 4000× *g*, and the pellet was resuspended in 10 mM MES (pH 5.5) and 10 mM MgSO_4_ to an OD_600_ of 0.2 and incubated for 3 h at room temperature. Bacterial cells were syringe- or vacuum-infiltrated into plant cells. Leaves were harvested 4 days after infiltration (dpi).

### 4.5. SDS-PAGE and Western Blot Analyses

For small-scale expression, pieces of infiltrated leaves (8–10 mg) were excised and homogenized in 50 μL of the PBS buffer (50 mM sodium phosphate pH 8.0, 300 mM NaCl). The resulting suspension was mixed with half volume of the 3× sample buffer (40% glycerol, 4% SDS, 50 mM Tris pH 6.8, 1% bromophenol blue, 5% beta-mercaptoethanol) resulting in total protein fraction for SDS-PAGE or was subjected to centrifugation at 14,000× *g* for 10 min. The supernatant, representing soluble protein fraction, was mixed with 3× sample buffer. Then, 10 μL of the obtained mixture (corresponding to about 1 mg of leaf tissue) was analyzed by SDS-PAGE and Western blotting. After electrophoresis, the gel was stained with One-Step Blue Protein Gel Stain (BIOTIUM, San Francisco, CA, USA) or was used for protein transfer onto a Hybond-P membrane (GE Healthcare, Chicago, IL, USA) by semi-dry transfer using the Trans-Blot Turbo Transfer System (Bio-Rad Laboratories, Hercules, CA, USA). After transfer membranes were applied to iBind Western System (Invitrogen, Waltham, MA, USA) following the manufacturer’s instructions. 

For Western blotting, we used monoclonal anti-influenza A M2e mAb 14C2 (Abcam) at a dilution of 1:10,000 (1 mg/mL) and monoclonal antibodies against RBD (Xema, Moscow, Russia) at a dilution of 1:10,000 (2 mg/mL). The rabbit anti-mouse antibodies conjugated with peroxidase (Promega, Madison, WI, USA) was used as the secondary antibodies at a dilution of 1:10,000. Specific protein–antibody complexes were visualized using a Western Blot ECL Plus kit (GE Healthcare, Chicago, IL, USA) and chemiluminescence detector Fusion Solo X (Vilber, Eberhardzell, Germany). The relative intensities of the bands in the digital photographs were analyzed using Nonlinear.Dynamics. TotalLab. TL120.v2009-NULL software. 

### 4.6. Isolation and Purification of Plant-Produced HEV/M2 and HEV/RBD Proteins

Purification of plant-produced proteins was performed using metal affinity chromatography under native conditions. For the purification, agroinfiltrated leaves of *N. benthamiana* were homogenized in PBS buffer (50 mM sodium phosphate, pH 8.0, 300 mM NaCl). The suspension was filtered through a paper filter and subjected to centrifugation at 14,000× *g* for 10 min; the supernatant was used as a soluble protein extract. The extract was mixed with Ni–NTA resin (QIAGEN, Hilden, Germany) equilibrated with PBS, and incubated for 60 min. The resin was then washed with PBS containing 10 mM imidazole (twice) and with 20 mM imidazole (twice). The elution was carried out in 500 mM imidazole. After elution, the protein was dialyzed against PBS (1:100, 4 buffer changes) using 3.5 K MWCO Slide-A-Lyzer Mini (Thermo Fisher Scientific, Waltham, MA, USA). The dialyzed protein preparation was passed through a 0.22 μm Sprintzen/Syringe-Filter (TPP Techno Plastic Products AG, Switzerland). Proteins were quantified using the Qubit Protein Assay Kit using the Qubit Fluorometer (Thermo Fisher Scientific, Waltham, MA, USA) according to the manufacturer’s instructions.

### 4.7. Nanoparticle Analysis

Analysis of particle size by dynamic light scattering method was performed using a Zetasizer NanoS90 particle size analyzer (Malvern).

Atomic force microscopy was performed using an Integra Prima microscope and Nova SPM software (NT-MDT, Moscow, Russia). The scanning was performed in semi contact mode using gold cantilever NSG01 (NT-MDT). 

Electron microscopy was performed on a JEM 1400 instrument (JEOL, Tokyo, Japan). Purified proteins were placed on carbon-formvar-coated copper grids (TED PELLA, Redding, CA, USA) and stained with 1% (*w*/*v*) uranyl acetate in methanol. The average size of the particles was determined using 10 particles.

### 4.8. Serum Samples and ELISA

Serum samples were collected from patients infected by SARS-CoV-2 (*n* = 24). The infection was confirmed by PCR test. Samples were taken after the full recovery of the patients. A total of 10 pre-pandemic (2016–2018) serum samples were used as negative controls to determine whether plant-produced HEV/RBD was able to recognize SARS-CoV-2 specific antibodies. The study was conducted in accordance with the Declaration of Helsinki and approved by the Institutional Ethics Committee (No. 7/17.05.16) of the Russian Medical Academy of Continuous Professional Education of the Ministry of Health of the Russia.

ELISA polystyrene plates (Nunc Medisorb, Thermo Scientific, Denmark) were coated with 12 μg/mL of the HEV/RBD or HEV proteins in 0.05 M bicarbonate/carbonate buffer (pH 9.5) and incubated for 16 h at 4 °C. After a single wash with PBST, the plates were incubated with 100 μL/well of blocking solution (1 mg/ml BSA in PBS) for 1 h at 37 °C. After a single wash with PBST, aliquots of sera of COVID-19 patients diluted 1:80 in blocking buffer (1% (*w*/*v*) BSA in PBS) were dispensed into the wells of the plates and incubated for 30 min at 37 °C. Control sera were used at 1:80 dilution. After five washes with PBST, peroxidase-labelled anti-human IgG (H + L) antibody solution (Vector-BEST, Novosibirsk, Russia) was added. After incubation with the conjugate for 30 min, wells were washed five times with PBST. Tetramethylbenzidine (Vector-BEST, Novosibirsk, Russia) was used as a substrate for horseradish peroxidase; the incubation time was 20 min. The reaction was stopped by adding sulfuric acid (1 M) and the optical density was measured at 450 nm in a StarFax 3200 spectrophotometer plate reader (Awareness Technology, Palm City, FL, USA).

## 5. Conclusions

Truncated genotype 3 HEV ORF 2 consisting of a.a residues 110 to 610 could be a promising VLP carrier platform for the presentation of foreign antigens. We demonstrated that expression of recombinant capsid protein of HEV consisting of a.a. residues 110 to 610 (HEV 110-610) bearing M2e peptide of influenza A virus or RBD fragment of SARS-CoV-2 spike glycoprotein at Tyr485 position in *Nicotiana benthamiana* plants is feasible. Both recombinant proteins, HEV/M2 and HEV/RBD, formed VLPs in vivo and could become promising candidates for the development of vaccines against influenza A or SARS-CoV-2. The ELISA showed that antibodies produced from recovering COVID-19 patients can bind plant-derived HEV/RBD, showing that the plant-produced material is immunologically relevant.

## Figures and Tables

**Table 1:** Nanosized structures formed by HEV-containing proteins.

Method	HEV	HEV/M2e	HEV/RBD
Atomic force microscopy	particles (26 ± 5 nm)	particles (60 ± 7 nm)	particles (40 ± 4 nm)
Electron microscopy	particles (18 ± 8 nm)	particles (42 ± 6 nm)	particles (33 ± 4 nm)
Dynamic light scattering	particles (25 ± 4 nm) aggregates ~100 nm	particles (48 ± 3 nm) aggregates (200–300 nm)	particles (42 ± 2 nm) aggregates (200–300 nm)

## Data Availability

Not applicable.

## Abbreviations

AMV	5′-nontranslated region of RNA 4 of the alfalfa mosaic virus
ER	endoplasmic reticulum
GFP	Green fluorescent protein
HEV	Hepatitis E virus
M2e	extracellular domain of membrane protein M2 of influenza A virus
PBS	Phosphate buffered saline
PVX	Potato virus X
RBD	Receptor Binding Domain of the S protein of SARS-CoV-2
SARS-CoV-2	Severe acute respiratory syndrome coronavirus 2
SP	signal peptide
VLP	virus-like particle
